# Insights in the Development and Uses of Alternatives to Antibiotic Growth Promoters in Poultry and Swine Production

**DOI:** 10.3390/antibiotics11060766

**Published:** 2022-06-02

**Authors:** Md Ramim Tanver Rahman, Ismail Fliss, Eric Biron

**Affiliations:** 1 Faculty of Pharmacy, Université Laval, Québec, QC G1V 0A6, Canada; ramimbau@gmail.com; 2 Laboratory of Medicinal Chemistry, CHU de Québec Research Center, Québec, QC G1V 4G2, Canada; 3 Institute of Nutrition and Functional Foods, Université Laval, Québec, QC G1V 0A6, Canada; ismail.fliss@fsaa.ulaval.ca; 4 Food Science Department, Faculty of Agriculture and Food Sciences, Université Laval, Québec, QC G1V 0A6, Canada

**Keywords:** growth promoters, alternatives to antibiotics, poultry production, swine production, acidifiers, bacteriophages, enzymes, phytochemicals, probiotics, antimicrobial peptides

## Abstract

The overuse and misuse of antibiotics has contributed to the rise and spread of multidrug-resistant bacteria. To address this global public health threat, many countries have restricted the use of antibiotics as growth promoters and promoted the development of alternatives to antibiotics in human and veterinary medicine and animal farming. In food-animal production, acidifiers, bacteriophages, enzymes, phytochemicals, probiotics, prebiotics, and antimicrobial peptides have shown hallmarks as alternatives to antibiotics. This review reports the current state of these alternatives as growth-promoting factors for poultry and swine production and describes their mode of action. Recent findings on their usefulness and the factors that presently hinder their broader use in animal food production are identified by SWOT (strength, weakness, opportunity, and threat) analysis. The potential for resistance development as well as co- and cross-resistance with currently used antibiotics is also discussed. Using predetermined keywords, we searched specialized databases including Scopus, Web of Science, and Google Scholar. Antibiotic resistance cannot be stopped, but its spreading can certainly be hindered or delayed with the development of more alternatives with innovative modes of action and a wise and careful use of antimicrobials in a One Health approach.

## 1. Introduction

The demand for animal-origin food products is still increasing today to meet the dietary needs of a growing world population and the rising financial capacities of the inhabitants of several countries who can now afford to purchase more animal proteins. This constant growth of the animal origin food products market has encouraged the extension of intensive farming worldwide to increase production and satisfy the increasing demand [1,2].

In recent decades, intensive animal husbandry practices have allowed increased yields, efficiency, and cost reduction [3], but often at the expense of animal welfare, the environment, and human health [4]. Because poultry and livestock are often kept under crowded conditions in intensive animal farming [5], infection transmission is favored, and the animals are more susceptible to diseases [6,7]. Most poultry and livestock diseases will affect animal health and decrease productivity, but some are transmissible to humans. To prevent diseases and the transmission of infections among animals, farmers use antibiotics that, if overused or misused, lead to the evolution of bacteria and the rise of drug-resistant pathogens in the long term [8,9].

It has been observed that the use of sub-therapeutic antibiotics in animal feed could significantly increase poultry and livestock productions [10,11,12]. The beneficial effects of sub-therapeutic antibiotics as growth promoters include the minimal occurrence of subclinical diseases, the reduction of morbidity and mortality, the increase of daily growth rate, the decrease of feeding costs (require 10–15% less feeding to achieve the desired level of growth), the maximal conversion of feed to animal product, and improvement of reproduction and meat quality (more protein and less fat) [13,14,15]. While some families of antibiotics used as growth promoters in poultry and livestock such as tetracyclines, penicillins, and aminoglycosides are also commonly administered to humans to treat bacterial infections, others such as ionophores are used only in animal husbandry. It was recently reported that most of the highest priority and critically important antimicrobials have relatively low sales volumes for human use [16]. However, a recent study on antimicrobials sales in 41 countries for chicken, cattle, and swine productions reported sales of 93,309 tons in 2017, and that number is projected to reach 105,000 tons by 2030 [17]. Another study in 2015 on cattle, chicken, and swine productions estimated that the global average annual intake of antimicrobials per kilogram of animal raised was around 45 ppm, 148 ppm, and 172 ppm, respectively [18]. In order to limit or eliminate the use of growth-promoting antimicrobials in agriculture, several countries have adopted action plans with a focus on antimicrobials that are significant in human medicine (Table 1). For example, China, the largest producer and consumer of antibiotics feed, used 162,000 tons of antibiotics in 2013, half of which were given to animals [19], and has now officially entered the period of “no antibiotics in feed” [20,21]. Regulations on the use of antibiotics as growth promoters can vary significantly from one country to another. For example, the use of the growth promoter bambermycin is allowed in Australia, New Zealand, and the USA, while it has been banned from all use in EU livestock since 2006 [22].

**Figure 1:**
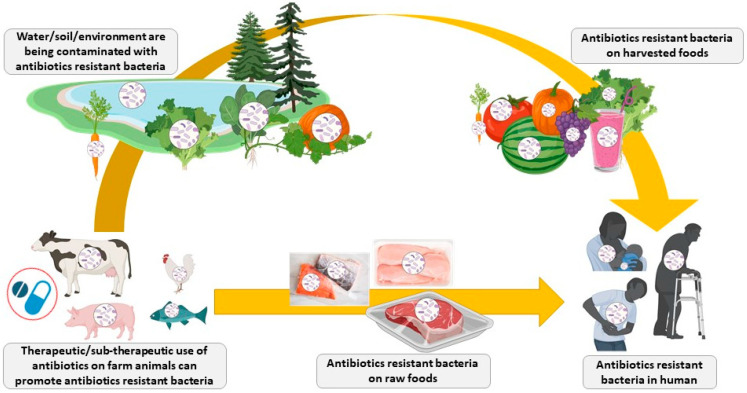
Spread of antibiotic-resistant bacteria from livestock animals to humans (Figure created in biorender, https://biorender.com/).

The overuse and misuse of antibiotics in agriculture and veterinary and human medicine led to the emergence of multi-resistant pathogenic strains and the spread of antibiotic resistance around the world [31,32]. Nowadays, infections by antibiotic-resistant bacteria cause more than 0.7 million deaths annually worldwide, but it is estimated that such infections could kill over 10 million people by the year 2050 if the antibiotic resistance crisis cannot be controlled [33,34]. According to the World Bank, antimicrobial-resistance may cost the world economy US$1 trillion annually after 2030 [35]. The increase of antibiotic resistance has also been observed in poultry and livestock farming worldwide [36] and contributes directly as well as indirectly to the rise of infections caused by antibiotic-resistant bacteria in humans (Figure 1) [37,38]. It is estimated that over 60% of established infectious diseases can be propagated from animals to humans (zoonotic diseases, also known as zoonoses) and that 75% of new or emerging infectious diseases in humans are from animals [39,40,41]. It was recently reported that in low- and middle-income countries, 13 poultry- and livestock-related diseases that can affect humans caused up to 2.4 billion cases of illness and 2.7 million deaths in humans per year [42]. Fortunately, the direct transmission of pathogens from poultry and livestock animals to human via food products is usually minimized, as proper food processing, handling, and cooking methods are destroying them in the food production chain. However, the most important concerns for the use of antibiotics as growth promoters in poultry and livestock farming are: (1) the use of sub-therapeutic amounts of antibiotics could promote the development of antibiotic-resistant strains through selection pressure; (2) the release of antibiotic-resistant bacteria in the environment; (3) the transfer of antibiotic-resistant genes to non-resistant bacteria in the environment or human flora by horizontal (processes of conjugation, transduction, or transformation) or vertical transfer; and (4) the release of small amounts (residuals) of antibiotics and their metabolites in the environment could promote de novo mutations or evolution of sensible bacteria, leading to antibiotic resistance [43,44,45,46,47].

With the removal of antibiotic growth promoters in food animal production policies and approaches in several countries in response to the growing global threat of antibiotic resistance, alternatives are now urgently required to prevent diseases and promote growth in food animal production. Several alternative approaches and products that do not contribute to a selection pressure to promote the development of antibiotic resistance have been studied and developed in the last decade, but some issues are hindering their extensive use in commercialization. This review article aims to describe the most promising alternatives to antibiotic growth promoters in poultry and swine production with a focus on their use, efficiency, and modes of action, including co- and cross-resistance profiles that are driving the evolution and spread of antibiotic resistance.

## 2. Modes of Action of Antibiotics to Promote Animal Growth

**Figure 2:**
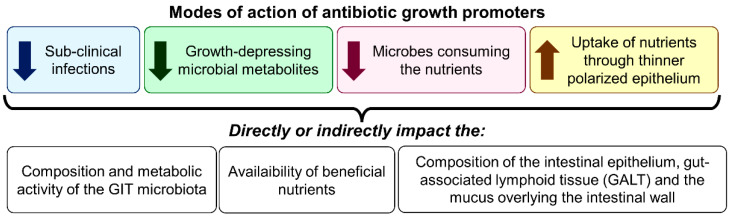
Proposed modes of action of antibiotics as growth promoters.

The application during the past few decades of several improved practices and knowledge in poultry and livestock farming, such as the introduction of high-growth and reproductive genetic selection, the use of innovative husbandry practices (hygiene, vaccination, shelter, mobility, etc.), and better understanding of the digestive physiology and dietary requirements of farm animals has resulted in substantial productivity gains [48]. The observation that the use of sub-therapeutic amounts of antibiotics in animal feed could significantly promote growth was certainly another breakthrough in poultry and livestock production. In human medicine, health is often linked to the “absence of clinical disease”. However, this definition cannot be extended to farm animals, as it is well-recognized that animal performance can be compromised without any clinical signs of disease [49]. This difference probably motivated farms to investigate the use of sub-therapeutic amounts of antibiotics in farm animal feed as disease preventers and growth promoters [50]. Different mechanisms are involved in controlling animal health and growth through the use of antibiotics, and some are still not well-understood [51]. Even if various physiological (digestion and absorption), nutritional (diet), metabolic, and immunological reactions to feed-grade antibiotics have been recorded, the common result is that their use increases feed efficiency and growth speed, even at constant feed intake. In pigs’ feed, growth responses are commonly related to enhanced apparent nitrogen digestibility (3% increase), increased nitrogen retention (5% increase), and reduced nitrogen excretion (10% decrease). Regardless of dietary protein content, growth-promoting antibiotics also improve protein metabolism. At least four modes of action have been proposed to explain the improved antibiotic-mediated animal growth: (1) the inhibition of sub-clinical infections; (2) the reduction of growth-depressing microbial metabolites in the intestines; (3) the increase of nutrient availability via the reduction of microbes sharing the nutrients in the intestines; and (4) the improvement of uptake and use of nutrients through thinner polarized epithelium (Figure 2) [52,53].

Commensal and pathogenic gut bacteria decrease animal development either directly or indirectly via their metabolic activities. Current evidence has diverged into two primary hypotheses: (i) bacteria-centric and (ii) host-centric. In the first, it is proposed that the antimicrobial activity of antibiotics can lower the population or diversity of the gut microbiota, reducing competition for nutrients and microbial metabolites that influence development (amino acids and bile catabolism). For example, bacteria in the small intestine tend to compete with the host for energy and amino acids. As a result of the bacteria’s consumption of glucose to create lactic acid, the host epithelium has less energy available for its operation. Others suggested that antibiotics decrease inflammation and the generation of pro-inflammatory cytokines, which decrease hunger and promote muscle catabolism [54]. The anti-inflammatory role of growth-promoting antibiotics reduces wasted energy and directs it toward production [51]. It is clear that a shift in microbiota composition (structure and diversity) does occur when antibiotics are included in animal diets [55]. These changes will eventually contribute to an optimal and balanced microbiota that is less likely to elicit an inflammatory response, increases the nutrient energy harvest, and allows animals to achieve their genetic potential. There is also evidence that bile salt hydrolase (BSH) enzyme producing bacteria are reduced by antibiotics [56]. In animal models, it was observed that low doses of antibiotics increase the copy number of genes involved in the metabolism of carbohydrates to short-chain fatty acids (SCFA) [57,58]. Based on this knowledge, researchers now work towards the recognition of specific bacterial populations that definitively enhance animal growth and the identification of approaches and resources to achieve a desired microbiota. Following the widespread use of antimicrobial growth promoters in animal feed and the observation of their impact on poultry and swine production, several parameters such as weight gain, feed conversion rate, intestinal morphology, microbiota composition, production of digestive enzymes, immune response, and carcass quality have been established as growth-promoting indicators (summarized in Table S1) [59,60,61,62,63,64,65,66,67].

## 3. Bacterial Resistance: Cross-Resistance and Co-Resistance

Cross-resistance and co-resistance can continue to promote antibiotic resistance in bacteria in the absence of antibiotics or antimicrobials. Cross-resistance is a single resistance mechanism that confers resistance to an entire class of antibiotics or different classes of agents such as the production of an efflux pump or an antibiotic-modifying enzyme. For example, the regulating protein CzcR controls the expression of the CzcCBA efflux pump in *Pseudomonas aeruginosa*, which confers resistance to zinc, cadmium, and cobalt. CzcR also co-regulates resistance to last-resort antibiotics carbapenems in *P. aeruginosa* by repressing the expression of OprD porin, the path these antibiotics use to cross the external membrane and reach the bacterial cell wall [68]. Co-resistance refers to the presence of resistance to two or many classes of antibiotics/substances in the same bacterial strain. It refers to the presence of several resistance genes on the same genetic material, such as on plasmid or transposon [69,70]. For example, co-resistance for amoxicillin and ciprofloxacin in *E. coli* indicates that using one of these antibiotics will increase resistance for both amoxicillin and ciprofloxacin at same time [71].

To reduce environmental impacts and limit the emergence and spread of resistance, it has now become essential to consider co- and cross-resistance during the design and development of alternatives to antibiotic growth promoters in food animal production. Among the most promising approaches developed to replace antibiotics as growth promoters, the most well-known and studied are the phytochemicals, acidifiers, probiotics, prebiotics, synbiotics, enzymes, bacteriophages, and antimicrobial peptides.

## 4. Alternatives to Antibiotics as Growth Promoters

### 4.1. Phytochemicals

In the field of phytochemistry, phytochemicals are chemical compounds produced by plants in an evolutionary process to acquire defensive systems against microbes, insects, animals, extreme temperatures, and ultraviolet (UV) irradiation. Also known as phytobiotics, phytogenics, herbals, or botanicals, phytochemicals are non-nutritive components to specific plants and parts of plants. Interestingly, whole plants or different parts of plants have been used in traditional medicine since the beginning of human evolution for the treatment of various diseases. Because of their antioxidant and anti-inflammatory properties, phytochemicals played and are still playing an essential role in the discovery and development of several drugs, according to preclinical, clinical, and epidemiological studies [72]. However, some phytochemicals can cause acute and chronic adverse effects, and even promote cancer [73]. The World Health Organization (WHO) developed and launched in 2013 “The WHO traditional medicine strategy 2014–2023” to quantify traditional and complementary medicine, including herbs and other plant materials [74]. The phytochemicals market for human and animal use was around US$834 million in 2014 and is expected to reach US$9 billion by 2029 [75].

#### 4.1.1. Modes of Action

**Figure 3:**
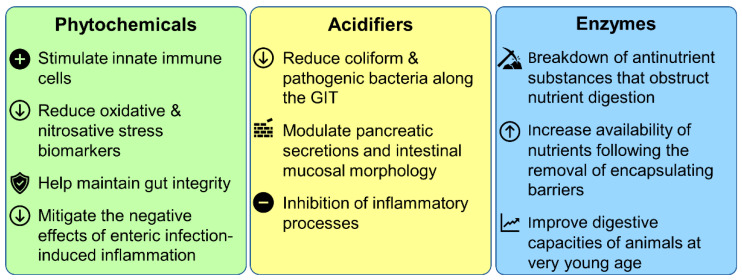
Modes of action of phytochemicals, acidifiers, and enzymes as growth promoters.

A wide variety of modes of action have been reported in the literature (summarized in Figure 3) [76,77,78,79]. Despite these observations, most phytochemicals’ modes of action are not fully understood. It is generally believed that their synergistic antimicrobial activity relies on their lipophilic properties and abilities to bind or damage membranes or to minimize cell division by inhibition of DNA synthesis. The efficiency of phytochemicals as antibiotic alternatives for improving animal growth performance has been demonstrated in chicken, swine, beef, and dairy production. Essential oils [80], oleoresins (solvent-free), and natural extracts are phytochemicals that are generally recognized as safe (GRAS) for their intended use [81,82]. The biological activities of phytochemicals were shown to be dose-dependent, having distinct physiological effects at varying doses. It was observed that the addition of phytochemicals (plant extracts) during the earliest phases of production, particularly in poultry and pigs, and the use of isolated phytochemicals during the last phase, notably in pigs and cattle, will have good impacts [58]. The use of an equal mixture of carvacrol and thymol as a feed additive in broiler chickens showed enhanced growth-promoting effects on performance, antioxidant enzyme activities, fatty acid composition, digestive enzyme activities, and immune response [83]. Moreover, it was observed that oregano and other herb extracts can suppress the growth of harmful coliform bacteria in broiler chickens without affecting the growth of beneficial bacteria [84]. In another study, the use of the natural polyphenol resveratrol in broiler chickens feed reduced more significantly the number of cecal *E. coli* than feed containing colistin [85]. In contrast, it was observed in another study that the integration of grape seed extracts in chicken diets up to 2500 ppm did not affect growth efficiency and digestibility of amino acids, but an increase to 5000 ppm led to a decrease in feed conversion and delay of growth rate [86]. This research also found that grape polyphenols suppress plasma-minerals. Another study with pigs showed that the addition of dietary chestnut tannin to pig diets (at 1.5–5.3 g/kg) has no effect on digestibility, nutrient use, or efficiency [87].

#### 4.1.2. Bacterial Resistance to Phytochemicals

Very few studies in the literature describe the effect of phytochemicals on the production of antibiotic resistance genes (ARG) and mobile genetic elements (MGE). In a recent study, the use of mushroom powder feed decreased the abundance of fecal *Roseburia* strains and favored the growth of *Campylobacter* strains compared to carbadox (antibiotic), CuSO_4_, and ZnO (metal). Fecal analysis showed that phytochemical-based growth promoters increased linkages between ARG and MGE (abundances of ARG and MGE) [88]. As with antibiotics, the critical problem is that bacteria can evolve and become resistant to the active phytochemical components over time.

#### 4.1.3. Strengths and Weaknesses

Several studies with phytochemical-based products have shown beneficial effects on animal growth performance, intestinal inflammation, and microbiota; however, some drawbacks such as bad odors, need of high doses to obtain results, and toxicity have been observed in some of them [89,90,91]. The SWOT analysis for phytochemicals is presented in Table 2. Despite the great potential of phytochemicals as animal growth promoters, a lot of research still needs to be done to ensure the highest level of results and to assess the timely introduction (phases of production), the phytochemical composition (blends or individual compounds), and the kind of active compounds suited for each type of animal. Better knowledge on the functional mechanism behind their physiological activity, the optimal dosage and duration regime, and their mode of administration for maximum benefit will certainly improve their efficiency and safety. From a chemistry point of view, the development of an extraction and isolation process to produce phytochemical compounds in high purity can be very challenging and expensive. Lastly, although phytochemicals are considered “natural” items, they should be deeply evaluated for potential detrimental human and animal health effects as well as probable interactions with other dietary elements [82].

### 4.2. Acidifiers

Acidifiers are organic acids such as benzoic, citric, formic, fumaric, lactic, and propionic acid or their salt counterparts such as calcium, potassium, or sodium formate or sodium fumarate [92,93]. With their lower cost, inorganic acids such as hydrochloric, sulfuric, or phosphoric acids have been considered as alternatives to organic acids, but their effects were different from most organic acids, as their mode of action is based on pKa values [94]. From a chemical point of view, most organic acidifiers bear one or several carboxyl (COOH) functional groups that play an important role in their activity and can also be found on amino acids and fatty acids. Few acidifiers can form complexes with minerals such as calcium (Ca^2+^) and zinc (Zn^2+^) cations that will reduce their absorption in the digestive tract. Acidifiers have been generally recognized as safe (GRAS) agents since 1972 and have been used in poultry diets and drinking water for decades with positive responses on growth performance [95,96,97]. The feed acidifiers market is estimated to grow from US$2.7 billion in 2018 to US$3.5 billion by 2023 [98].

#### 4.2.1. Modes of Action

All acidifiers have some level of antibacterial effects. Three types of modes of action have been identified for acidifiers: the reduction of coliform and pathogenic bacteria, the modulation of pancreatic secretions and mucosal morphology, and the inhibition of inflammatory processes (Figure 3) [92,99,100]. A study analyzed the effects of a commercial acidifier containing formic, propionic, and acetic acids combined with cinnamaldehyde on *salmonellosis* in laying hens and found that acidifier supplementation can manipulate immune response and decrease *Salmonella* infection in laying hens [101]. In another study, it was observed that butyric-acid-containing feed (organic acid blend supplementation) reduced the total number of *S. typhimurium* compared to a control group [102,103]. Some acidifiers may also act as energy sources and help to reduce the tissue wastage resulting from high rates of gluconeogenesis and lipolysis [104]. Additionally, short-chain fatty acids have been shown to promote proper crypt cellular proliferation, increasing tissue regeneration and maintenance [105]. Organic acids can also show antiviral, antifungal, and antimold properties [106].

The beneficial effects of acidifiers on animal health and growth has been recently reviewed by Tugnoli et al. for swine production [94] and Khan et al. for broiler and layer chickens [99]. Most reported studies observed that the use of acidifiers in animal feed reduces microbial intestinal colonization and infectious processes in addition to having an inhibitory effect on inflammatory processes at the intestinal mucosa [107]. Overall, this led to improved villus width, height, and area of the duodenum, jejunum, and ileum of broiler chicks, as well as secretion, digestion, and nutrient absorption [108,109,110]. With the intention of improving delivery and performance of acidifiers, some commercial acidifiers have been encapsulated by fatty acids or other molecules to allow their controlled release in specific compartments of the intestines [111]. Unfortunately, the modes of action of acidifiers have not been studied in depth until now, and further research is needed to determine their mechanisms of action, behavior in animals, and the optimal conditions for their use and best results.

#### 4.2.2. Bacterial Resistance to Acidifiers

Comparable to other antibiotics, organic acids show a broad spectrum of antimicrobial activity [112] which can contribute to the expression of the antibiotic resistance gene (ARG) and mobile genetic element. Interestingly, a study in pigs did not find any associated genes encoding resistance to tetracycline, streptomycin, or sulfonamide when feed with a blend of propionic, formic and acetic acids, cinnamaldehyde, and a permeabilizing complex was used [113]. Another study showed that, compared to feed containing the antibiotic enrofloxacin, acidifier-based feeds (formic, propionic, and acetic acids) did not change the total number of cecal *E. coli*. However, these acidifier-based feeds significantly decreased the population of *E. coli* resistant to ampicillin, tetracycline, sulfamethoxazole, and ciprofloxacin [114]. It was also showed by Ngoc et al. that a basal feed containing a mixture of formic, acetic, lactic, propionic, citric, and sorbic acids, ammonium formate, and a combination of medium-chain fatty acids (C8, C10, and C12) inhibited the apparition of multidrug-resistant *E. coli* strains (resistant to amoxcillin/clavulanic, cefotaxime, ceftiofur, ciprofloxacin, nofloxacin, and flumequine) [115].

#### 4.2.3. Strengths and Weaknesses of Acidifiers

Besides their low cost and the possibility of producing them on a large scale, the main advantages of acidifiers include the reduction of the retention of indigestible food in the intestine, inhibition on pathogenic microflora, and greater preservation of animal feed [116,117,118]. However, most acidifiers still show some weaknesses; the addition of acidifiers at an extreme level can negatively affect diet palatability, feed manufacturers can observe corrosiveness, which is harmful for feed processing equipment, and further research is needed to improve quality control and optimal dosage and to allow a better understanding of the potential threats [92,116,119]. The SWOT analysis for acidifiers is presented in Table 3.

### 4.3. Enzymes

Feed additive enzymes are biologically active proteins which enable the breakdown of specific chemical bonds of nutrients into smaller compounds for further digestion and absorption. Phytase, carbohydrases, xylanase, α-galactosidase, β-mannanase, α-amylase, β-glucanase, proteases, lipases, and pectinase are some of the most commonly used feed enzymes. Interestingly, the enzymes used in animal feed are commonly produced by bacteria, fungi, and yeast such as *Bacillus subtilis* for α-amylase, *Trichoderma reesei* for cellulase, *Aspergillus niger* for β-glucanase, and *Saccharomyces cerevisiae* for invertase [120]. Exogenous enzymes can increase gut stability by reducing substrates for putrefactive organisms, increasing substrates for beneficial fermentative organisms, and improving the intestine’s ability to protect itself against unwanted bacterial condensation [121]. Post-weaning diarrhea is one of the most severe risks to the global swine industry. The antibiotic colistin is commonly used in pigs for the oral treatment of *E. coli*-related intestinal infections, particularly post-weaning diarrhea [122]. Exogenous β-mannanase has been shown to decrease post-weaning diarrhea without compromising gut health or overall efficiency [123]. Positive effects have also been obtained in swine with the use of corn–soybean meal diets supplemented with 0.1% β-mannanase [124]. A study showed that up to 10% of dry residue of cassava can be used in broiler diets from 21 to 42 days of age when associated with carbohydrase for performance maintenance [125]. A synergistic effect of carbohydrases (xylanase and β-glucanase) used with phytase and/or an acidifier composed of formic acid, propionic acid, lactic acid, ammonium formate, and ammonium propionate reduced *E. coli* counts and increased villus length in broiler chickens [126]. The global feed enzyme market has been estimated at US$103.8 billion in 2020 and is predicted to reach US$144.1 billion by 2025 [127]. On a global basis, the use of carbohydrases, phytases, and proteases saves the animal feed industry more than US$8 billion per annum in nutritional input costs and helps limit the environmental impacts [128].

#### 4.3.1. Modes of Action

High substrate specificity adds a specific feature to an enzyme. Each enzyme recognizes specific substrates and performs their modification at specific reaction sites [129]. The proposed modes of action for feed enzymes include: (i) the breakdown of antinutrient substances that obstruct nutrient digestion; (ii) the increased availability of nutrients following the removal of the encapsulating barrier; and (iii) the improvement of digestive capacities of animals at a very young age (Figure 3) [129,130]. However, the observed animal response to food enzymes is influenced by factors like feed humidity levels, dietary pH, the length of time required for enzymes to interact with the substrate, and many more [129].

#### 4.3.2. Bacterial Resistance to Enzymes

Enzymes can have direct antimicrobial effects by hydrolyzing bacterial cell walls or compromising the glycocalyx’s integrity. The lysozymes are a group of most well-known antimicrobial enzymes that can hydrolyze the peptidoglycan in bacterial cell walls and cause cell death [121,131].

#### 4.3.3. Strengths and Weaknesses of Enzymes

The greatest strength of enzymes is their ability to increase digestibility and nutrient availability while degrading antinutritional factors [129,132]. Enzymes reduce feed costs and improve feeding efficiency, but enzyme-based products often suffer from poor quality control and lack of information about their concentration and optimal use conditions [121]. Another factor that limits the value of enzymes is that they are formulated at a fixed dose while the majority tend to release nutrients in a log dose: linear nutrient release relationship [128]. The SWOT analysis for enzymes is presented in Table 4.

### 4.4. Probiotics and Direct-Fed Microbials (DFM)

Probiotics are defined as “live microorganisms which are administered in adequate amounts to confer a health benefit on the host”. Probiotics can be bacterial (*Lactobacillus*, *Bifidobacterium*, *Bacillus*, and *Enterococcus*) or non-bacterial (yeast and fungal) and allochthonous (normally not present in the intestines flora of animals) or autochthonous (indigenous organisms of the intestines flora of animals). Probiotics are occasionally administered to animals who have been treated therapeutically with antibiotics or other medications to allow recolonization or reinforcement of the gut flora that may have been weakened or depopulated during the therapy. Most studies have demonstrated that administering probiotic strains alone or in combination greatly boosts the average daily feed intake (ADFI), average daily gain (ADG), and feed conversion ratio (FCR) in pigs and poultry [133,134]. However, several studies observed no difference in carcass yield, growth rate, or feed utilization efficiency of birds treated with a commercial probiotic like GalliPro^®^ [135]. The conflicts in results could be related to differences in probiotic strains and/or the bird breeds tested [136]. Exopolysaccharides (EPS) retrieved from probiotic bacteria can be used as prebiotics in poultry and swine production. However, because EPS from probiotic bacteria have shown anti-inflammatory, antibacterial, and anti-oxidant activities [137] and an ability to regulate chicken intestinal microbiota [138], further investigation is needed. According to Markets and Markets, the global market for probiotics in animal feed is expected to grow from US$4.6 billion in 2019 to US$7.0 billion by 2025 [139]. In comparison, the global probiotics market by application (functional food and beverages, dairy products, non-dairy beverages, infant formula, cereals, dietary supplements, feed), ingredient (bacteria, yeast), and form (dry, liquid) is expected to reach US$69.3 billion by 2025. Custom-designed probiotics by genetic engineering can play a vital role to find out the fittest probiotics for animals.

#### 4.4.1. Modes of Action

The use of probiotics in animal feed has been shown to increase the population of beneficial microorganisms such as *Lactobacillus* and *Bifidobacterium* species through the production of lactic acid and SCFA and reduction of pH and to inhibit the growth of harmful microorganisms such as *Campylobacter jejuni* or *Salmonella enteritidis* through the release of inhibiting substances like organic acids [140] and/or bacteriocins [141]. Another key effect of probiotics is the modification and regulation of bowel immune responses through a reduction in pro-inflammatory cytokines and an increase of IgA production and promotion of specific and non-specific immune responses to pathogens (activation of macrophages, cytokine production by intraepithelial lymphocytes) [51]. It was also demonstrated that some probiotics can improve nutrient digestion and absorption by increasing the structure of the crypts and villus height in the intestines. To that end, *Bacillus subtilis* is a widely utilized bacteria that has been shown to increase intestinal villus height [142]. Some probiotic bacteria can also improve digestive capacities of the host by producing enzymes. For example, *Bacillus licheniformis* strains have been used because of their abilities to produce amylase, alkaline proteases, β-mannanase, and keratinase, which are effective for broilers’ growth [143,144].

#### 4.4.2. Bacterial Resistance to Probiotics

The use of probiotic strains in animal feed has raised several questions and concerns about the risks of emergence of acquired antibiotic resistance in bacteria present in the intestinal microflora. Since probiotic strains contain genes for immunity to some antimicrobials and antibiotic resistance, they could transmit antibiotic resistance genes to pathogenic bacteria through horizontal gene transfer [145]. To minimize this risk, it is important to verify if a prospective probiotic strain contains potentially transferable resistance genes. For example, a study with sixteen *Lactobacillus* isolates from chicken and calves intended for use as probiotics showed four isolates displayed resistance to tetracycline and aminoglycoside antibiotics, while others were susceptible to a large panel of 15 different antibiotics [146]. PCR analysis of the identified resistant *Lactobacillus* isolates confirmed the presence of resistance genes. The propagation of antibiotic resistance through probiotics has been summarized by Daniali et al. [147]. For optimal results, a proper variety of probiotic strains must be evaluated. Safety assessment protocols for a probiotic candidate have been established to limit the different risks associated with the use of probiotics in animal feed [148].

#### 4.4.3. Strengths and Weaknesses of Probiotics

Most studies on the use of probiotics in animal feed have reported a wide variety of beneficial effects on animal growth and health. Along with the beneficial impact on gut microbiota and inflammation, it was observed that probiotics can reduce diarrhea and improve feed digestion by producing enzymes or by promoting digestive enzyme secretion in the intestines [60,134,142,149]. However, several concerns with some probiotic-based products such as variations in the quality and dose of probiotics, poor survival rate in the stomach, inactivation during feed manufacturing, transport, or storage, allergenicity, potential crosstalk between probiotics, pathogens and epithelial cells, and transmission of antibiotic-resistance genes can limit their use [140,147,149,150]. The SWOT analysis for probiotics is presented in Table 5. Probiotics have shown very promising results as alternatives to antibiotics in animal feed, and further research will allow a stronger product quality control, determination of the optimal dosage for the target animal, and a better understanding of their impact on different physiological functions of the target animal on antibiotic-resistance transmission and on the environment.

### 4.5. Prebiotics

Prebiotics are compounds that act like food components or fertilizer for beneficial microorganisms in the gut by stimulating their growth. Prebiotics include a wide range of non-starch polysaccharides or oligosaccharides such as mannan-oligosaccharide, fructans (fructooligosaccharide and inulin), galactans (galacto-oligosaccharide), malto-oligosaccharide, lactulose, lactitol, and gluco-oligosaccharides. These nondigestable oligosaccharides are fermented in the large intestine by beneficial bacteria and provide energy for the microbiota [153,154]. Some dietary fibre types can be considered prebiotic as well [155]. A study on the use of prebiotics in swine feed showed that the addition of a galacto-oligosaccharide mixture inhibited the attachment of enterohepatic *E. coli* and *S. enterica* subtype typhimurium to HT29 cells and increased the number of *Bifidobacterium* and *Lactobacillus* in stool [156]. Similar results were obtained with poultry, where the use of fructooligosaccharide [157], chicory fructans [158], fructan-rich Jerusalem artichoke, or topinambur [159] improved the activities of amylase and total protease and increased the number of *Lactobacillus* in the small intestine. It was also observed that male bird counts of *Campylobacter* and female bird counts of *Salmonella* were lowered, and the levels of endotoxins in the blood were reduced in male and female boiler chickens. Another study concluded that some detrimental effects of heat stress could be reduced by the prebiotics [160].

Even the meat quality traits of chicken (lower redness index, lightness and yellowness not affected) are positively affected by the use of prebiotics [161]. A recent review on the prebiotic effects of seaweed polysaccharides demonstrated that they may be used to promote pig health throughout the production cycle, hence lowering antibiotic use [162]. The role of prebiotic supplementation in improving growth performance, immunological regulation, and pathogen reduction has been studied extensively and reviewed in depth by Adhikari et al. [163].

#### 4.5.1. Modes of Action

Prebiotics are neither digested nor absorbed in the upper intestines, but serve as a food supply for beneficial bacteria found in the lower intestines, such as *Lactobacillus* (LAB) and *Bifidobacterium* [163]. Animal enzymes cannot degrade prebiotics in the intestines [164]. They have been hypothesized to act via inhibiting pathogen adhesion, immunomodulation, fermentation-based synthesis of antimicrobial chemicals, and alteration of gut morphology [165]. Some sugars can block the binding of pathogens to the mucosa. Prebiotics are considered eco-friendly, but their use and regulation are not well-established [166].

#### 4.5.2. Bacterial Resistance to Prebiotics

Our search did not find any literature about the effect of prebiotics on ARG and mobile genetic elements, as prebiotics themselves are unable to inhibit and kill microorganisms.

#### 4.5.3. Strengths and Weaknesses of Prebiotics

Most reported prebiotic-based products in animal feed did not show antimicrobial activity by themselves, but their use promoted few beneficial bacterial strains in gut and inhibited the growth of some pathogenic strains [163,167]. Despite their beneficial effects on the intestine, such as increased villi height and lower pH, the administration of a large amount of prebiotics might induce unwanted side effects such as bloating or diarrhea due to the fermentation in the intestines [149,168,169]. The SWOT analysis for prebiotics is presented in Table 6.

### 4.6. Synbiotics

Synbiotics are combinations of probiotics and prebiotics developed to circumvent some of the challenges associated with probiotic survival in the intestines. Taking advantage of the probiotics and prebiotics characteristics, synbiotics have been shown to have a greater effect on the microbiota than probiotics or prebiotics used separately, with enhanced production of lactic acid and SCFAs and a reduction in BCFAs concentration [170,171]. The benefits of synbiotics go beyond the improved growth and microbiota health; they also include the limitation of antibiotic resistance development. For example, broilers challenged with a multi-resistant *E. coli* strain that received an organic acid-based feed additive containing a mixture of formic, acetic, and propionic acids with cinnamaldehyde or a synbiotic preparation containing a combination of *Enterococcus*, *Pediococcus*, *Bifidobacterium*, and *Lactobacillus* strains with inulin did not yield a significant increase of antibiotic-resistant *E. coli* strains. In comparison, treatment of the same broilers with the antibiotic ampicillin led to a significant increase in the abundance of *E. coli* strains resistant to ampicillin, amoxicillin-clavulanic acid, cefoxitin, and ceftriaxone [172].

#### Modes of Action

The mechanisms by which synbiotics affect the host include the prebiotic that encourages the growth of probiotic bacteria or the prebiotic and probiotic bacteria that function independently in the intestines. To increase and modulate the intestinal microbiota, prebiotics (non-digestible substances) are fermented in the intestines by probiotic bacteria that colonize the intestinal space [173]. It has been shown that synbiotics can increase the count of beneficial bacteria and restrict the growth of potential pathogens in the intestines of broiler chickens [171,174,175]. However, although probiotic and synbiotic supplementation can positively modulate the intestinal microbiota, a study demonstrated that they were not effective in reducing *Salmonella* Typhimurium load in caecal tissue and invasion into vital organs such as liver and spleen in chickens [176]. Synbiotics can also affect the immune system of the host. A study in broiler chickens showed that early *in ovo* treatment with prebiotics and synbiotics modulates the production and maturation of leukocytes [177]. In another study, the use of a combination of the *Bifidobacterium breve* probiotic and GOS prebiotic significantly enhanced the defense against fatal intestinal infections caused by multidrug-resistant *Acinetobacter baumannii* in a mouse model [178]. Given the enormous variety of potential combinations, the use of synbiotics in animal feed to promote growth and modulate gut microbiota appears very promising.

As synbiotics are a mixture of prebiotics and probiotics, they have the same strengths and weaknesses as probiotics and prebiotics as well as the same potential risks for bacterial resistance development. Like pre- and probiotics, synbiotics reduce diarrhea, increase digestibility and daily weight gain, and promote beneficial bacterial strains, such as *Lactobacillus* and *Bifidobacterium* strains, leading to a more balanced gut microbiota [179]. The presence of prebiotics in the mixture assists probiotics in overcoming potential survival challenges [180]. However, the majority of synbiotics used in animal feed have insufficient probiotic/prebiotic mixing ratios, and appropriate controls would need to be used in experiments for the development of symbiotic-supplemented animal feed [181].

### 4.7. Bacteriophages

Bacteriophages are viruses that can infect only bacterial cells and kill their host by causing cell lysis. Although they were discovered in the beginning of 19th century, bacteriophages have attracted a lot of attention in recent years due to their excellent specificity, non-toxicity, and natural abundance. While phages have been exploited in eastern Europe for decades, they are still not yet well-accepted in the United States or other nations. This might be due to public concerns about elective viral use, problems with commercial phage manufacturing, or a lack of funding for and validation of clinical trials [182]. Bacteriophages are composed of proteins that form a capsid (head) and a tail and of DNA or RNA as the viral genome. While the capsid encapsulates and protects the genetic material, the tail is a complex multiprotein structure that plays a critical role in bacterial host recognition, attachment, digestion, cell wall penetration, and genome ejection. Initially, phages bind to bacteria and deposit their genome inside the host to eventually replicate in the cytoplasm until the infected cell is lysed. Afterward, the released virions can infect other bacteria in the environment. As a result, bacteriophages have a direct impact on bacterial populations [183,184].

Several studies on the use of bacteriophages to prevent infections in animals and humans to pathogens have yielded promising results. For example, the use of a cocktail of four bacteriophages exhibited activity against bovine and human *E. coli* O157:H7 isolates and was proposed for on-farm therapy [185]. It was also reported that bacteriophage biocontrol can reduce *Campylobacter jejuni* levels in chickens without affecting collateral effects on gut microbiota and help prevent human exposure and food-borne illness from contaminated poultry products [186]. Another study showed that bacteriophages infecting *Salmonella gallinarum* could be a promising alternative to antibiotics for the control of fowl typhoid disease in chickens [187]. The use of bacteriophages in the poultry industry has been discussed broadly by Żbikowska et al., and many studies supporting their great potential as alternatives to antibiotics are reported. However, further research is necessary to better understand specific phage–bacterium interactions, pharmacodynamics, and mechanisms of coevolution between phages and bacteria [188].

#### 4.7.1. Modes of Action

Bacteriophages have been shown to influence innate and adaptive immunity through phagocytosis and cytokine responses. By affecting the stability of the intestinal microbiota, bacteriophages can modulate the intestine’s immunological and metabolic capabilities. Their ability to affect the formation of bacterial communities by changing the parasitic or lytic phase of bacterial cells has also been described. Bacteriophage may aid bacterial colonization and survival in various anatomical places, particularly the commensal population’s ability to defend against diseases [189,190,191]. A study on the use of a dietary bacteriophage supplementation in weaned piglets showed the tested diet was able to promote growth performance through a positive effect on intestinal inflammation, intestinal barrier function, and gut microbiota with an enhanced number of *Lactobacillus* and *Bifidobacterium* bacteria [192].

#### 4.7.2. Bacterial Resistance to Bacteriophages

Bacteriophages and phage-like particles play important roles in bacteria horizontal gene transfers (HGT) and significantly contribute to their adaptation (short-term) and evolution (long-term). Given their abundance and varied DNA-packaging mechanisms, bacteriophages are attractive vehicles for the acquisition, maintenance, and dissemination of ARGs [193]. Twelve distinct forms of ARGs and the class 1 integron-integrase gene *intl1* were discovered in bacteriophage DNA fractions isolated from chicken feces, reinforcing the evidence that bacteriophages are essential ARG reservoirs in the world [194]. The presence of the *mcr-1* gene in bacteriophage DNA was also discovered for the first time in the same samples. In the same study, the absolute abundances of the *blaCTX-M* and *mcr-1* genes in bacteriophage DNA fractions were similar to or even higher than plasmid DNA [194]. A similar profile has been observed in swine, where bacteriophage DNA was present in 35.5 percent of the target ARG groups in pig feces. ARGs such as *sul1*, *blaTEM*, and *ermB* genes were found in 100% of the bacteriophage DNA samples, while *ermB* and *fexA* were the most abundant ARGs in the bacterial population [195]. A very interesting study on the contribution of bacteriophages to antibiotic resistance demonstrated that viromes from non-human sources such as pig feces, raw sewage, and freshwater and marine environments contain a large reservoir of ARGs, while human-associated viromes rarely carry ARGs [196]. Bacteria can acquire resistance from lysogenic phages containing sequences encoding bacterial resistance or toxins in their genetic material and begin to acquire such resistance after incorporation of the phage’s genetic material into the bacterium genome. In addition to these processes, bacteria can hydrolyze the phage’s genetic material by restricting endonucleases found in their cytoplasm and can methylate their own DNA as a phage protective mechanism. The results point out that phages could play a part in the spread of antibiotic resistance. Bacterial resistance to bacteriophages may also be caused by gene mutations encoding proteins that are either important for phage reproduction or required to assemble new virion particles [197].

#### 4.7.3. Strengths and Weaknesses of Bacteriophages

Bacteriophages have unique characteristics. The immune response is not their priority as it is for vaccines, they do not leave residual compounds in the environment as antibiotics or chemicals do, and they do not indirectly influence the microbiota as probiotics do. Since bacteriophages have a very narrow spectrum of activity and target specific problematic strains without altering the microflora, their mode of action as growth promoters is mainly via their antimicrobial activity [197,198]. Several challenges associated with their production, stability, regimen, and risks of antibiotic resistance transmission still limit their use in animal feed [149,199]. The SWOT analysis for bacteriophages is presented in Table 7. More research is still needed to determine the optimal dosage, frequency, and formulation and better understand their impact on target animal growth and antibiotic resistance development.

### 4.8. Antimicrobial Peptides

Antimicrobial peptides (AMP), also known as host defense peptides, are an important family of short amphipathic proteins (less than 100 amino acids) which constitute part of the innate immune defense existing in nearly all classes of organisms. Of the approximately 5000 currently known linear and cyclic AMPs, most are cationic (positively charged) [154]. AMPs are often broad-spectrum inhibitors of Gram-positive or Gram-negative bacteria, but some, e.g., bacteriocins produced by bacteria, can exhibit narrow spectra of activity. It is also well-established that most AMPs are innate and adaptive immune effector molecules that can modulate pro- and anti-inflammatory responses and chemotactic activity [155]. With an 80-year application history, AMPs are considered strong candidates to replace antibiotics in the animal food production industry and have been widely studied [201]. For example, broiler feed supplemented with the bacteriocin microcin J25 improved performance, reduced systemic inflammation, improved fecal microbiota (lower population of total anaerobic bacteria), and decreased *Salmonella* infection rate [202]. Similar beneficial effects have been observed with the use of AMPs in piglet feed [201,203,204,205]. The results shows that AMPs can be powerful antibiotic substitutes, especially under infection conditions [201,203,204,205].

#### 4.8.1. Modes of Action

The antibacterial activity of most AMPs is based primarily on the interaction of positively charged peptides with negatively charged components of the bacterial membrane such as phospholipids and teichoic acids of Gram-positive bacteria or lipopolysaccharide of Gram-negative bacteria, which leads to pore formation, membrane permeabilization, and cell lysis after re-localization in the cytosolic membrane. Membrane permeabilization may also result in the translocation of specific AMPs into the cytoplasm, where they inhibit main cellular processes such as DNA and protein functions or synthesis [206]. Although the antimicrobial activity of AMPs plays an important role in their impact on animal growth, their ability to modulate the immune response also strongly contributes to their beneficial effects. It has been shown that supplementation of feed with microcin J25, a bacteriocin active against several *E. coli* and *Salmonella* strains, can promote growth performance, improve intestinal morphology, influence fecal microbiota composition, and reduce the secretion of pro-inflammatory factors (IL-1β, TNF-α, IL-6) in broilers [202]. It was also observed that microcin J25 was able to attenuate intestinal inflammation diseases caused by enteric pathogens [207]. In another study with crude recombinant piscidin, the use of AMP-supplemented feed in farmed chickens increased weight gain, feed efficiency, and production of IL-10 and IFN-γ [208]. Beneficial effects of AMPs in swine nutrition on performance, nutrient digestibility, intestinal morphology, and intestinal and fecal microflora have also been observed [209,210]. The use of colicin-supplemented feed on weaning pigs yielded a 40% higher weight gain and 7% lower feed efficiency [211]. In another study, Wu et al. showed that a diet containing a chimeric cecropin AD was able to improve growth performance and reduce the incidence of diarrhea in pigs [212]. Although most AMPs did not provide equal effects to that of antibiotics in swine nutrition, several studies show their great potential as an alternative for antibiotics in rations fed to swine [209,210].

#### 4.8.2. Bacterial Resistance to Antimicrobial Peptides

Resistance to AMPs can be either innate (intrinsically found in particular genera, species, or strains) or acquired (developed by a formerly susceptible strain), and both can be linked to several genetic loci in bacteria. Since AMPs exert their activity through a great variety of mechanisms, multiple strategies have been developed by microorganisms to counter their action [213,214]. Resistance mechanisms involving the secretion of proteases or peptidases, modification of the cytosolic membrane permeability, lipid composition, or electric potential, alteration of the target, and downregulation of target gene expression have been reported [215]. As with antibiotics, there is a risk that bacteria can evolve and become resistant to the used AMP over time. However, it has been observed that the frequency of genes undergoing spontaneous mutation upon cellular exposure to low concentrations of AMPs and resulting in AMP resistance is low [216,217]. Since AMPs and antibiotics act via very different modes of action, the development of cross-resistance between AMPs and antibiotics is believed to be rather limited. Nevertheless, increased resistance to some antibiotics was observed in variants resistant to an AMP [218,219]. For example, Mantovani and Russell observed a 1000-fold increase in resistance to ampicillin in nisin-resistant mutants of *Streptococcus bovis* compared to the original nisin-sensitive isolates [220]. The effects of the exposure to AMP on the development and spread of resistance are not yet fully understood, and this is something that will need to be carefully studied and monitored if AMPs are used in animal feed.

#### 4.8.3. Strengths and Weaknesses of Antimicrobial Peptides

Numerous studies on the use of antimicrobial peptides as growth promoters have shown their great potential as alternatives to antibiotics. Their abilities to improve growth performance and gut health, positively influence the microbiota, decrease the occurrence and severity of diarrhea, and inhibit the expression of pro-inflammatory factors have been observed [221]. In addition, the degradation of antimicrobial peptides in the intestines prevents their release into the environment and reduces the risk of exposure that can lead to the development of resistance. However, this force is also a weakness, as it decreases the half-life of the peptides in the intestine. Despite these attractive characteristics, the use of peptides has heretofore been limited by the problems associated with their large-scale production, their stability during feed preparation and storage, and their interactions with feed matrices [210,222]. The potential development of resistance and cross-resistance with clinically important antibiotics is also an important feature to investigate in depth. The SWOT analysis for antimicrobial peptides is presented in Table 8. Other interesting characteristics of peptides include the possibility to produce them via fermentation or chemical synthesis, to easily perform modifications to improve their stability and their activity, and to use different formulations to improve their bioavailability in the gut [223]. There is still more research to be done on the use of AMPs as growth promoters in animal feed in order to improve their performance and beneficial impact in different animal species, to evaluate synergistic effects with other alternatives, and to better understand their modes of action on animal growth and the development of resistance and cross-resistance.

## 5. Regulation and Approval of Alternatives to Antibiotics for Use in Animals

**Figure 4:**
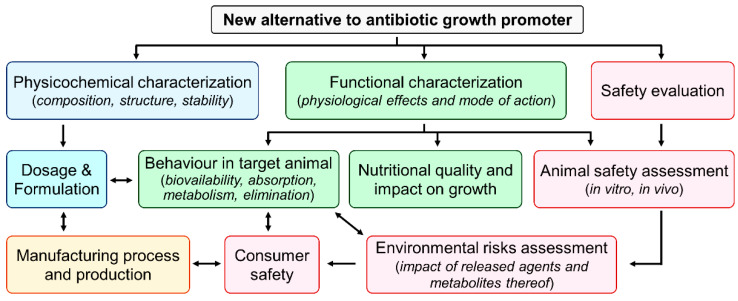
Important parameters to assess for alternatives to antibiotic growth protomers mixed with feed ingredients before large-scale use.

Bringing a new alternative to antibiotic growth promoters to market involves assessing its safety for the animal, consumer, user, and environment as well as its efficacy, acceptability, and feasibility (Figure 4). At the end, a variety of criteria determine whether a certain alternative is successfully commercialized or not. For example, overall costs and benefits, regulatory approval, and target animals are important criteria [224]. The regulatory approval varies from country to country, but the analysis process remains essentially the same. The alternatives outlined in this manuscript are based on unique technologies with modes of action that have not yet been subjected to regulatory examination, demanding significant assessment. The speed of innovation is fast, and it encompasses a wide range of items that do not always meet the traditional definition of a veterinary medical product or fit neatly into any existing product categories, necessitating clarity on the regulatory framework and criteria that should be applied to them [225].

**Figure 5:**
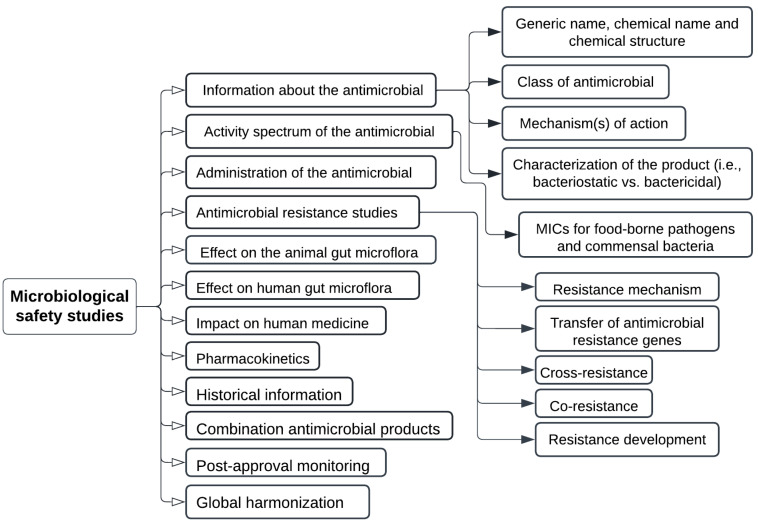
Microbiological safety assessment for alternatives to antibiotic growth promoters as per regulation approval, where cross resistance, co-resistance, resistance gene transfer, and resistance development mechanism are categorized as antimicrobial resistance studies.

Generally, a veterinary drug can take 7–10 years to come to market. Regulation of animal drugs and nutritional supplements in the United States is managed by the Center for Veterinary Medicine of the United States Food and Drug Administration (FDA). There are three different types of new animal drug applications: (i) NADA, used to approve a novel animal drug; (ii) ANADA, used to obtain a generic new animal drug; and (iii) CNADA, used to seek conditional approval of a new animal drug. Veterinary drugs in Canada are regulated by The Food and Drugs Act and Regulations administered by Health Canada, which ensures the safety and effectiveness of the product. The Feeds Act and Regulations govern animal feeds in Canada and are administered by the Canadian Food Inspection Agency (CFIA), whose objective is to assure the safety, effectiveness, and correct labelling of livestock feeds under the feeds act, feeds regulations, health of animals act, health of animals regulations, and organic products regulations. There are many different types of mixed feeds that may be produced by combining various ingredients. There shall be no mixing of components that have not been approved for use in any mixed feeds. If a new antibiotic alternative is used in feed ingredients, the following assessment (Figure 4) is required by the CFIA and microbiological safety data sheet (Figure 5), and it should be reported as antibiotic alternative if antibacterial effects are also observed.

## 6. Discussion and Conclusions

Taken together, a wide range of products and new formulations are now being developed and tested to replace the use of antibiotics as growth promoters in rations feed for poultry and swine. It is expected that the ongoing quest for more functional and sustainable alternatives will continue to increase the portfolio of target functions that will be subjected to further research. While no alternative so far can claim to replace antibiotics fully in animal feed, several of them have considerable value and may be part of practical ‘antibiotic-free’ poultry and swine production programs. Even though some alternatives to antibiotics have been shown to help animals grow and stay healthy, the main problems with most of them are how reliable they are, how different they are between species, how much they cost, and how hard they are to make. For example, since the intestine physiology and microbiota vary from one animal breed to another, an alternative may work better in one animal breed than in another. A suitable alternative dosage in the feed-in function of animal breeds and species will also be critical to obtain the best results and avoid side effects. A better understanding of the digestive systems and gut microbiota of animal species and breeds will undoubtedly help determine the optimal dosage of the developed alternatives in animal feed. Ingredient quality and content knowledge is another essential concern, as contaminants that are not listed or tested could substantially impact the results [223]. The purity of enzyme products for use in animal feeds is rarely shown. Allergenicity has not been addressed so far and would be another critical function to investigate with the help of alternatives to antibiotics, as allergic reactions could affect performance and lead to serious inconvenience. There are also very little data on their general pharmacological properties, such as absorption in intestines, plasma half-lives, and toxicity. This knowledge is critical, as it will allow a better understanding of the behavior and fate of the used alternative products and their long-term impact on animals and the environment. For example, a product with a long half-life could accumulate in the body if it is absorbed or be released intact into the environment if it is not degraded in the intestines, increasing the risk of resistance development. Animal toxicity mechanisms and toxic doses are also poorly understood. A product in animal feed could also affect reproduction efficiency and fetal development, show neurotoxicity, or even cause inflammation, DNA strand breaks, chromosomal damage, or gene mutations. The potential transfer of alternative products such as phytochemicals, organic acids, and antimicrobial peptides into edible animal products and their potential harm to consumers should also be investigated.

It is also important to note that the intestines have a diverse range of nutritional and physicochemical conditions. In animals, growth performance is closely linked to intestine functions, many of which are conducive to the development and survival of biofilms. These biofilm-forming communities are not well-understood in terms of their nature, function, and involvement in pathophysiology and animal health [121,224,225]. As biofilm growth and abnormalities are most often associated with gastrointestinal illnesses, the impact of antibiotic substitutes in animal feed on biofilm formation and survival should also be considered and investigated during the development of alternatives to antibiotics.

As there does not appear to be an alternative product that can entirely replace antibiotics as a growth promoter in animal feed for poultry and swine production at this time, a combination of approaches is more likely to produce the necessary breakthroughs in this field. A combination of alternatives to antibiotics seems to be a good way to combat drug resistance, but it is not without flaws, as numerous key pharmacological problems remain unsolved. As we push forward with the overarching goal of minimizing the usage of antibiotics as growth promoters, special attention should be paid to sustainable manufacturing, environmental impact, probability of resistance development, the genetics of resistance evolution, and the threat of antibiotic cross-resistance during the development and regulation of alternatives to antibiotics in animal feed in poultry and swine production.

## Figures and Tables

**Table 1:** Regulations regarding the use of antibiotics as growth promoters in different countries.

Country	Year	Action
Australia	2017	Antibiotics used in human medicine are not licensed as growth promoters. However, five antibiotics (olaquindox, avilamycin, bambermycin, monensin, and salinomycin) not currently used in human medicine are used as growth promoters in poultry, pigs, cattle, and sheep [23].
Canada	2020	Growth promotion claims on medically important antimicrobials (MIAs) (Category I, II, and III antimicrobials) will no longer be permitted. Ionophore and coccidiostat products will be unaffected, as they are not considered MIAs [24,25].
China	2020	All antibiotic growth promoters except herbal medicine have been banned [26].
European Union	2006 2022	Illegal across the EU The EU will ban the importation of meat and dairy produced using antibiotic growth promoters. However, fluoroquinolones continue to be licensed in the UK and in many EU countries [27].
New Zealand	2017	No banning claim found. The Ministry of Health and the Ministry for Primary Industries (MPI) stated in 2017: “*If antibiotics used in food-producing animals, MPI must also be satisfied that the antibiotic will not leave residues above the maximum residue level in food from the treated* *animals*”. Compared to EU countries, New Zealand uses more cephalosporins and macrolides, but less quinolones [28].
Sweden	1986	First country to ban the use of antibiotics as growth promoters [29].
USA	2017	Medically important antimicrobials are banned. However, bacitracin and carbadox, which are classified as medically important by the World Health Organization, are still used as growth promoters in pigs [30].

**Table 2:** SWOT analysis of phytochemicals.

Strengths	Weaknesses [89]
Improve growth performance Reduce the markers of intestinal inflammation Maintain mucosal integrity	Very large dose may be needed to obtain results The lipophilic nature of some phytochemicals can limit delivery to enteric pathogens Can produce toxicity or other adverse effects The absorption and distribution of essential oils in the body might influence the organoleptic quality of animal products due to their high odoriferous qualities Phytochemical-supplemented feed can have a bad smell
**Opportunities** [89,90]	**Threats** [89,91]
Microencapsulation for targeted release can help Combination of essential oils with either disruptive metals, antibiotics, and/or nanotechnologies (synergistic effect) Dietary polyphenols can stimulate the growth of beneficial microorganisms in the intestines and an increase in the production of SCFA	Bacteria may adapt and become resistant to the active phenolic components Acquired resistance to phytochemicals is a transmissible plasmid function Can have effect on reproduction

**Table 3:** SWOT analysis of acidifiers.

Strengths [116,117,118]	Weaknesses [92,116,119]
Can be produced on a large scale Chelation of minerals Stimulatory effects on intermediary metabolism Can reduce the buffer capacity of feed and modulate the pH in the intestines Buffering acids (e.g., ammonia) make the product less hostile to the digestive system and mixing and feeding systems Intestinal retention of undigested feedstuff is reduced Can limit the proliferation of pathogenic microflora	Costly Often show inconsistent results that strongly depend on dose and time, diet composition, animal age, and environmental conditions The usage of certain acids may also be subject to legal restrictions. For example, pure formic acid is illegal in the United States, although formic acid salts are accessible for use in feeds
**Opportunities** [118]	**Threats**
Blends of acids Acidifier production, integrating coating, buffering, and microencapsulation A formulation with multicomponent acid composition may show a stronger effect Preservation of feedstuffs	Over time, bacteria may adapt and become resistant Effect on reproduction Extreme dosage may damage the esophagus and stomach

**Table 4:** SWOT analysis of enzymes.

Strengths [129,132]	Weaknesses [121]
Typically valued for their effect on feed cost reduction Can increase nutrient digestibility and availability of amino acids and minerals (especially multi-enzyme mixtures) Can degrade anti-nutritional factors	Activity of the enzyme is low Production and quality control standard is not high Enzyme release dynamic techniques rely on the nature of operation, diet composition, ambient temperature, and pH, among other variables. Knowledge of substrate concentrations is not adequate High level of acidity in the swine gut may inactivate in-feed enzymes
**Opportunities**	**Threats**
Selective enzyme for young and adult animal In most cases, high dosages do not have an adverse impact on output	Beneficial gut bacteria may die Can promote pathogenic bacteria

**Table 5:** SWOT analysis of probiotics and direct-fed microbials.

Strengths [134,142]	Weaknesses [140,149]
Diarrhea and intestinal trouble are avoided Produce organic acids Enhance feed digestion by producing enzymes (phytases, lipases, amylases, proteases) or promote digestive enzyme secretion by stimulating the intestines Improve the chemical, nutritional and sensorial characteristics of meat Some probiotic strains can survive in severe environments (stomach acid and bile acid)	Uncertainty about the quality of probiotics is noted from time to time, as is animal poisoning and allergic reactions following the use of probiotics Probiotics may be hazardous to animals born with a compromised immune system. Bacterial formulations can be easily inactivated during feed preparation, transport, and storage During use, most bacteria cannot withstand low pH in intestines and bile acids. It is difficult to get enough live cells to colonize the gut With the lack of proper related laws and standards, probiotic-based products cannot be labeled with the appropriate dose and suggested optimal dosage for the target animal, or other characteristics that may impact efficacy
**Opportunities** [151,152]	**Threats** [147,150]
Multistrain probiotic microorganisms are used to prevent newborn diarrhea It can bind and eradicate several targets, e.g., aflatoxin, aluminum, arsenic, cadmium, or lead, with feces Probiotics isolated from animals’ and humans’ intestines are safer for human and animal intake and may be more effective inside the intestinal environment	Probiotics can upset the natural balance of the microflora in the gut and other organs. For example, *Lactobacillus* and *Bacillus* can disrupt the ecological balance of the body’s natural flora, which may have a role in the development of urinary tract infections and other illnesses Crosstalk between probiotics, pathogens, and epithelial cells Gut microbiome is highly correlated with several mental disorders Probiotic bacteria may transmit antibiotic resistance genes and promote acquired antibiotic resistance

**Table 6:** SWOT analysis of prebiotics.

Strengths [163,167]	Weaknesses [149]
Promote beneficial bacterial strains such as *Lactobacillus* spp. and *Bifidobacterium* spp. Inhibit pathogenic strains, particularly, *E. coli* and *Salmonella* spp. Lower intestinal pH Increase villi height Increase immunity in gut-associated lymphoid tissues (GALT), increased number of IgG and IgM	Because of the fermentation in the intestines, feeding a lot of prebiotics might induce bloating, diarrhea, and other side effects
**Opportunities** [90]	**Threats** [149]
Some dietary polyphenols can work as prebiotics	Prebiotics are unable to prevent or treat bacterial infections, as they are unable to suppress and kill microorganisms by themselves

**Table 7:** SWOT analysis of bacteriophages.

Strengths [197,198]	Weaknesses [149,199]
Time is the key when it comes to phage therapy. Using phages early in the course of a disease could improve the therapeutic efficacy Research in animals and humans have reported the use of bacteriophages without altering the microflora Narrow and specific spectrum of activity	One of the primary challenges to eliminating pathogens from chickens is the necessity for large numbers of phages to adsorb individual host cells Problems associated with the manufacturing and stabilization of pharmaceutical preparations Preventive therapy did not prevent colonization
**Opportunities** [200]	**Threats** [149]
Cocktail of two or more bacteriophages to prevent the emergence of bacteriophage resistance	Bacteriophages may be able to transfer their DNA (pathogenicity determinants and virulence factors) from one bacterial cell to another, leading to resistance Lytic bacteriophages can be converted into lysogenic bacteriophages under specific conditions. Optimal dosage, route of administration, frequency, and treatment duration are still to be determined Toxic substances can be released by bacteriophages

**Table 8:** SWOT analysis of antimicrobial peptides.

Strengths [221]	Weaknesses [210,222]
Promote nutrient digestibility, gut health, and improved growth performance Modulate gut microbiota in a positive way and improve immune functions in the intestines Decrease the occurrence and severity of diarrhea Inhibit the expression of pro-inflammatory factors in the intestines Maintain mucosal integrity Thermostability Their rapid degradation in the intestines prevents the release of active AMP in the environment and reduces the risk of exposure leading to resistance development Easy degradation in environment	Production yields are usually low Chemical synthesis can be costly Susceptibility to oxidation during feed preparation and distribution Low resistance to proteolytic degradation by digestive enzymes resulting in short half-lives in the intestines Can react or interact with other compounds in the feed matrix, decreasing their bioavailability. Interactions with the feed matrix throughout product preparation can lead to structural change and inactivation of the AMP
**Opportunities ** [223]	**Threats** [210]
Peptidomimetics can be used to increase protease stability, stability in feed matrix, and activity Can be used with organic acids and/or phytochemicals to increase activity and beneficial effects (synergistic effects) Enteric formulation can increase stability in upper intestines and bioavailability in the gut	Can show high cytotoxicity Development of resistance and cross-resistance with clinically important antibiotics

## Supplementary Materials

The following supporting information can be downloaded at: https://www.mdpi.com/article/10.3390/antibiotics11060766/s1, Table S1: General parameters and growth promoting indicators.
