# Supplementary material for: Insights in the Development and Uses of Alternatives to Antibiotic Growth Promoters in Poultry and Swine Production
Source: Antibiotics (Basel). 2022 Jun 2;11(6):766. doi: 10.3390/antibiotics11060766 (PMC9219610; doi:10.3390/antibiotics11060766)
Supplement: Supplementary file 1 [file antibiotics-11-00766-s001.zip › antibiotics-1708657-supplementary.pdf]

antibiotics

Supplementary Material

Table S1. General parameters and growth promoting indicators.

Food animal	Characteristics	Observation[a]
**Broiler** [59-63]	Colonisation of *Campylobacter jejuni* and *Salmonella enteritidis* in the GIT	-
	Weight gain	+
	Bone characteristics, intestinal morphology	+
	Immune response	+
	Lysozyme and T lymphocytes	+
	Production of phytases, lipases, amylases and proteases (stimulate the GIT to secrete digestive enzymes)	+
	Vitamins, exopolysaccharides and antioxidants	+
	Pro-inflammatory and anti-inflammatory reactions	+/-
	Regulate pathogen numbers in the GIT	+/-
	Plasma metabolites of blood	-
	Triglyceride, total cholesterol, high-density lipoprotein (HDL), or low-density lipoprotein (LDL)	+
	LDL:HDL ratio	-
	Feed conversion ratio (g feed/g gain)	-
**Swine** [64-67]	Average daily gain (ADG)	+
	Feed conversion rate (FCR)	+
	Faecal microbial count: *Lactobacilli* and *Enterobacteriaceae* cell counts	+
	Histological analysis: Small intestinal tissues (duodenum, jejunum, and ileum), villous height (VH), crypt depth (CD)	+
	Carcass quality	+

[a]"+" indicates improvement, enhancement, increase; "-" indicates reduction, negative, decrease
